# Invasive Pulmonary Aspergillosis in a Haematopoietic Stem Cell Transplant Recipient with Sickle Cell Disease: a Successful Treatment

**DOI:** 10.4084/MJHID.2015.006

**Published:** 2015-01-01

**Authors:** Katia Paciaroni, Gioia De Angelis, Cristiano Gallucci, Cecilia Alfieri, Michela Ribersani, Andrea Roveda, Antonella Isgrò, Marco Marziali, Ivan Pietro Aloi, Alessandro Inserra, Javid Gaziev, Pietro Sodani, Guido Lucarelli

**Affiliations:** 1 International Centre for Transplantation in Thalassemia and Sickle Cell Anaemia, Mediterranean Institute of Haematology, Policlinic of “Tor Vergata” University, Rome, Italy; 2 Departments of Surgery, Ospedale Pediatrico Bambino Gesù, IRCCS, Rome, Italy.

## Abstract

Sickle Cell Anaemia (SCA) is the most common inherited blood disorder and is associated with severe morbidity and decreased survival. Allogeneic Haematopoietic Stem Cell Transplantation (HSCT) is the only curative approach. Nevertheless the decision to perform a bone marrow transplant includes the risk of major complications and transplant-related mortality. Infections represent the leading cause of death in SCA patients undergoing HSCT. Invasive Pulmonary Aspergillosis (IPA) is a devastating opportunistic infection and remains a significant cause of morbidity and mortality in HSCT recipients. Data regarding IPA in the setting of SCA are lacking. In the present report, we describe a patient with SCA, who developed IPA after allogeneic bone marrow transplant. The fungal infection was treated by systemic antifungal therapy in addition to surgery, despite mild chronic graft versus host disease (GVHD) and continuing immunosuppressive therapy. This case shows that IPA occurring in bone marrow recipients with SCA can be successfully treated.

## Introduction

Sickle Cell Anaemia (SCA) is a hereditary anaemia and a multisystem chronic disease that reduces the quality of life and increases mortality significantly since the third decade of life.^1^ Allogeneic Haematopoietic Stem Cell Transplantation (HSCT) is the only definitive cure for SCA. The decision to perform a bone marrow transplant (BMT) in SCA patients involves careful weighing of individual patient’s risk and benefits. The transplant-related mortality for SCA patients is approximately 7%, similar in all series of studies, and infections constitute the leading cause of death.^2^–^3^ Invasive Pulmonary Aspergillosis (IPA) is a possible infective complication and is a known cause of attributable mortality in HSCT recipients endangering their successful treatment (4). Data regarding the optimal management of IPA in transplanted SCA patients are lacking. Here we describe the first case of successful treatment of IPA in a sickle cell anaemia patient, recipient of bone marrow transplant (BMT).

## Case Report

A 17-year-old Nigerian boy with a diagnosis of SCA disease and recurrent episodes of vaso-occlusive crisis was referred to our centre as candidate to related allogeneic BMT. According to our internal protocol aimed at evaluating the possible lung damage of SCA patients before the transplant, computed tomography (CT) scan of the lung and spirometry were performed. The CT scan revealed no signs of infection and the spirometry test documented a restrictive pattern with a basic forced volume vital capacity of 79% and a not reached peak expiratory flow (64%). The condition regimen adopted for the HLA-identical related allogeneic transplant included: fludarabine (30mg/m^2^/day for 5 days), busulfan (16.48 mg/kg for 4 days) and cyclophosphamide (200mg/kg for 4 days). The graft-versus-host-disease (GVHD) prophylaxis consisted of intravenous cyclophosphamide, short-course methotrexate, prednisolone, and cyclosporine. Because of neurological toxicity signs on day +4 after transplant, cyclosporine was replaced initially by mycophenolate and then by the combination mycophenolate and tacrolimus (day +26). The neutropenic period lasted until the day +18 when the absolute neutrophil count was > 500 cells/mm^3^. The hospital stay after transplant was 32 days. Throughout the BMT pre-engraftment phase and the early post-transplant phase fluconazole was administered as the primary prophylaxis therapy. Microbiological cultures of sputum and of oral, nasal and pharyngeal swabs and serum galactomannan assay were weekly performed as surveillance.

**Figure 1:**
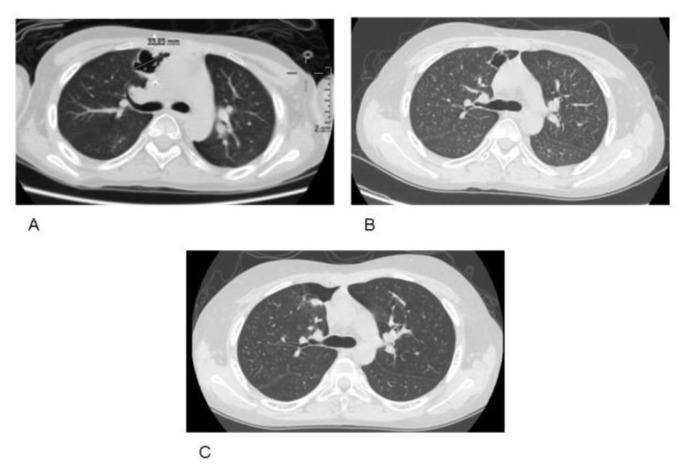
Lung computed tomography CT scan image: (**A**) large paracardiac rounded cavitary lesion (diameter 3 cm) in the right upper lobe, consistent with invasive pulmonary aspergillosis (IPA). (**B**): persistence of the cavitary lesion after five months of antifungal therapy (**C**) Complete disappearance of pulmonary aspergillosis, with a fibrotic lesion in place of the previous cystic lesion.

On day +40 after transplant, a II degree skin GVHD was diagnosed and immunosuppressive treatment was intensified by the use of multiple immunosuppressive agents (mycophenolate, tacrolimus and methylprednisolone 2 mg/kg). The acute GVHD was ameliorated and evolved in a mild chronic GVHD controlled with mycophenolate associated with low dose of steroids (0.5mg/kg) from day +122 after transplant. At day +83 after transplant, the first positive galactomannan test was detected (index 0.78, normal <0.5). The general condition of the patient was fair, and a mild productive cough without fever was the only symptom. A lung CT scan was performed and revealed a large pericardial rounded nodule (diameter 3 cm) with cavitation in the right upper lung lobe, suggestive of invasive aspergillosis (see Figure 1, A). In the same time, *Aspergillus terreus* was isolated from a sputum microbiological culture. The minimum inhibitory concentration (MIC μg/ml) of individual antifungal agents was determined by the methods described by European Committee for Antimicrobial Susceptibility Testing (EUCAST). The MICs of itraconazole and posaconazole against the *A. terreus* were 0.5μg/ml and 0.125μg/ml respectively, and were reported as susceptible (S). The MIC of Voriconazole was 0.03g/ml and was accompanied by the comment Insufficient Evidence (IE), according to epidemiological cut-offs established by the EUCAST. The amphotericin B was not tested against the *Aspergillus terreus* as EUCAST indicates for this species. Antifungal treatment with intravenous itraconazole at a dosage of 200 mg/d was promptly administered, according to the available antifungal susceptibility testing. Control lung CT scan performed one month after the initiation of the antifungal treatment showed minimal nodule size reduction (diameter 2.26 cm). However, the *Aspergillus* galactomannan antigen decreased and became negative 12 days after the initiation of treatment. After 42 days, intravenous itraconazole was switched to oral posaconazole. No hepatic toxicity was documented during itraconazole treatment, but the patient reported severe nausea and loss of appetite. Seven months after transplant (5 months after the antifungal therapy initiation), the immune system was partially recovered as documented by the immunophenotyping test (on day +209 after transplant the T-cells populations measured by flow cytometry were: CD3+ 1807/mm^3^, CD4+ 420/mm^3^, CD8+ 1301/mm^3^ CD19+ 240/mm^3^, CD16+CD56+ 578/mm^3^). Nevertheless, the control CT lung scans showed the persistence of the unmodified pulmonary lesion (Figure 1, B). As a consequence, in order to prevent possible pericardial erosion of the paracardiac pulmonary lesion, surgical therapy was considered. At day +226 after transplant, lung surgical segmentectomy was performed. The post-operative course was complicated by pneumothorax with prolonged air leak (14 days) treated with the use of a Bülau chest tube. Hospital stay was 22 days. Histological analysis of the excised tissue revealed an abscess cavity delimitated by fibrosis with a massive inflammatory reaction replacing the normal pulmonary parenchyma and numerous hyphae and fungal spores. Microbiological culture of the tissue also resulted in the growth of *Aspergillus terreus*. The lung CT scan performed 42 days after surgery documented the presence of a fibrotic replacement of the previous cystic lesion. (Figure 1, C). The patient was discharged and could go back to Nigeria maintaining a single-drug immunosuppression therapy and secondary prophylactic antifungal treatment with voriconazole (the only triazole available in his country). One year after transplant the immunosuppression therapy and antifungal treatment were discontinued. To date, 20 months after transplant, no evidence of recurrent fungal infection has been documented.

## Discussion

At present, allogeneic BMT represents the only chance for a cure of SCA.^1^ This procedure, provided that a matched healthy sibling donor is available, may represent the only option for long-term survival of most SCA patients living in developing countries, where the supportive therapy is often not provided. Nevertheless, the decision to perform BMT is not an easy one due to the associated risk of major complications and mortality. In the setting of SCA, the transplant-related mortality is, constantly across different studies, 7%.^2^–^4^ Of note, infections represent the primary cause of transplant-related mortality in SCA patients.^2^–^4^ Systemic fungal infections, especially IPA, are significant complications and a significant cause of morbidity and mortality in the transplant setting. Patients with acute leukemia, solid organ transplant recipients, and HSC recipients account for 29%, 9% and 32% of all *Aspergillus* infections, respectively.^5^ Data from prospective trials indicate that the attributable mortality of all disease entities range between 30% and 40%.^6^–^8^ Invasive aspergillosis has been well characterized in adults for the setting of transplant for malignant diseases.^4^,^9^ Yet, its incidence, risks factors, outcome and optimal treatment have not been extensively investigated in pediatric patients affected by hemoglobinopathies undergoing BMT. Among allogeneic HSCT recipients, three moments of risk for invasive aspergillosis occur, during the neutropenic phase following the conditioning regimen, the acute GVHD and the chronic GVHD. In particular the timeline of IPA in these patients follows a bimodal distribution, with a initial peak in the first month following HCST, associated with neutropenia, and a second peak during the treatment of GVHD (median 78–112 days post-transplant).^4^,^9^ Several others factors predispose transplanted patients to develop IPA: multiple immune defects, parenteral nutrition, use of various antibiotics, prolonged hospitalization, patient’s underlying conditions, chronic lung disease.^4^ In our patient case, a complete screening was performed before transplantation, in order to investigate the possible underlying pathologies. The spirometry test showed a decline in the lung volume with a restrictive pattern, probably attributable to pulmonary SCA complications. Indeed it is known that pulmonary disease in SCA has both acute and chronic components, and chronic lung disease is characterized by both parenchymal and vascular abnormalities.^10^ In the described SCA patient, the transplant was uncomplicated in the early phase, with short pre-engraftment neutropenia, short hospitalization, no parenteral nutrition and no use of multiple antibiotics. Conversely, the IPA occurred during the intensive immunosuppressive treatment for GVHD including use of various drugs. Probably, in a patient with a no malignant disease like SCA the short pre-engraftment neutropenia is less risky compared to the phase of multi-drug anti-GVHD therapy. Once IPA is developed, a positive outcome depends on early diagnosis, prompt initiation of adequate antifungal therapy and immune-system recovery. Despite the GVHD and the related immunosuppressive treatment, the immune system of the reported SCA patient was recovered, as assessed by the immunophenotype tests, and probably this immune reconstitution contributed to control the IPA infection. Monitoring with microbiological and serial galactomannan tests was useful for an early diagnosis and prompted for timely therapy initiation. Until a few years ago amphotericin B was considered the preferred drug. The field of antifungal agents for aspergillosis has expanded markedly in recent years with the development of several classes of mould-active agents. These include several new generation triazoles, echinocandins and less toxic formulations of amphotericin B.^4^,^11^ Recently voriconazole has became the gold standard as primary therapy for invasive aspergillosis, however both voriconazole and amphotericin B are the only compounds licensed for the primary treatment of invasive aspergillosis in the United States.^11^ In the present case, the mould specie was the *Aspergillus terreus*, an uncommon but emerging fungal pathogen which causes lethal infections and is often refractory to amphotericin B.^12^ Indeed in this case, the selection of the antifungal agent was made not empirically following the international recommendations but according to the antifungal susceptibility test results which reported the susceptibility only for itraconazole and posaconazole. Therefore, initially, the treatment was itraconazole administered intravenously to guarantee the optimal absorption and then switched to oral posaconazole, a broad-spectrum triazole. The systemic drug therapy probably prevented the dissemination of the infection but failed to completely eradicate the lung lesion. Since the penetration of antifungal agents into an area with a large cavitary lesion may be suboptimal, the resection of the area was considered. Surgical resection has generally a controversial role in the management of patients with IPA: neutropenia, thrombopenia and poor general conditions may increase perioperative morbidity and mortality and the redeeming benefit is questionable. However the efficacy and safety of surgical intervention, in addition to antifungal therapy has been demonstrated and the surgical resection is usually indicated for patients with solitary and persistent lung lesion and in case of haemopthysis or pulmonary high risk region (i.e. affecting the pericardium or to the great vessels).^13^ Nevertheless there is no randomized, prospective trial for optimized treatment including the antifungal and surgery approach. In the described case, the pulmonary lesion had a para-cardiac location, close to pericardium, and the patient was expected to receive prolonged immunosuppressive therapy because of chronic GVHD. Such a combination of risks, prompted us to plan surgical removal. The intervention was effective and safe. IPA was eradicated as documented by the radiological tests. Surgical intervention in addition to drug therapy has shown efficacy and was safe in this SCA patient despite mild chronic GVHD and with continuing immunosuppressive therapy.

## Conclusion

IPA is a serious infective complication affecting the outcome of HCST and remains a considerable diagnostic and therapeutic challenge. Its role in patients with haemoglobinopathy disorders requiring BMT is less known. In the present study, we report the successful management of a SCA patient who developed post-transplant IPA by adequate systemic anti-fungal therapy in addition to surgery. Further studies are needed on the epidemiology and the best therapeutic approach for IPA in this setting.
